# Validity and reliability of the persian version of the chronic oral mucosal diseases questionnaire

**DOI:** 10.22088/cjim.9.2.127

**Published:** 2018

**Authors:** Atena Shirzad, Ali Bijani, Mahsa Mehryari, Mina Motallebnejad, Saman Mohsenitavakoli

**Affiliations:** 1 Department of Oral Medicine, Faculty of Dentistry, Babol University of Medical Sciences, Babol, Iran.; 2 Social Determinants of Health Research Center, Health Research Institute, Babol University of Medical Sciences, Babol, Iran.; 3 Cellular and Molecular Biology Research Center, Health Research Institute, Babol University of Medical Sciences, Babol, Iran.; 4 Department of Oral Medicine, School of Dentistry, Babol University of Medical Sciences, Babol, Iran.

**Keywords:** Chronic Oral Mucosal Disease, Reliability, Validity, Quality of life, Questionnaire

## Abstract

**Background:**

Chronic oral mucosal disease** q**uestionnaire (COMDQ) is used to evaluate the quality of life in patients with chronic conditions of the oral mucosa. The aim of the present study was to evaluate the validity and reliability of the Persian version of this questionnaire.

**Methods:**

A total of 135 subjects were selected in two groups; group 1 consisted of 95 patients with chronic oral mucosal conditions, including recurrent aphthous stomatitis, oral lichen planus and pemphigus and mucous membrane pemphigoid and group 2 consisted of 40 patients with other oral diseases. The subjects completed the demographic data sheets and COMDQ and then underwent examinations to determine disease severity. After 14 days, the questionnaire was completed again by the group 1subjects only.

**Results:**

Cronbach’s α coefficient for COMDQ was estimated at 0.969 and the interclass correlation coefficient was estimated at 0.997. There was a significant relationship between the mean COMDQ scores and disease and pain severity. There was a clear correlation between the patients’ self-report about their general health and mean COMDQ scores and also between their opinions about their oral health and the mean COMDQ scores.

**Conclusions:**

The Persian version of COMDQ exhibited proper levels of reliability and validity. It is suggested that this questionnaire be used for the evaluation of the effect of treatment on the oral health-related quality of life (OHQoL).

Chronic oral mucosal disease comprises a wide range of relatively common disorders in the oral soft tissues that arise under autoimmune, inflammatory or infectious states (1). Recurrent aphthous stomatitis, oral lichen planus, pemphigus vulgaris, mucous membrane pemphigoid and oral granulomatosis are examples of these disorders. Chronic oral conditions are rarely life-threatening; however, they can significantly disrupt patients’ physical activities, social and psychological states due to their chronic, painful and recurrent nature and the long-term management periods, symptomatic treatments involved and also the side effects of treatment (2‒4). Therefore, the use of quantitative and observational parameters is not sufficient for the evaluation of these conditions and their symptomatic treatments; rather, adjunctive tools should be used to evaluate how these conditions and their treatment affect the daily activities of patients and their quality of life. These tools have been termed life quality assessment tools and are completed by the patients to see the condition from the patient’s point of view. The patient is in fact interviewed in relation to his/her status with the use of such questionnaires. This way the subjective conditions (the clinical manifestations) of the disease are more accurately evaluated through objective criteria so that it would be possible to evaluate the treatment needs of the patient, make proper decisions for the treatment of the patient and achieve good clinical results (5‒7).

Life quality questionnaires are used in three forms: discipline-specific, generic and disease-specific, of which the discipline-specific type is the most, appropriate one. This type of questionnaire is highly accurate and sensitive in relation to clinical changes of the patients and can predict clinical related changes (8‒11). COMDQ (chronic oral mucosal disease questionnaire) is a discipline-specific questionnaire that evaluates the quality of life in patients with chronic mucosal conditions and was first presented by Ni Riordain in Ireland in 2011. This questionnaire consists of 26 questions in 4 domains of pain and functional limitations, medication and treatment, social and emotional status and patient support. The reliability and validity of this questionnaire have been confirmed (4, 11-13). Due to variations in the social and cultural structures of different communities, OHQoL tools should be adapted to different cultures and languages to preserve the integrity of the questionnaire and extract reliable data from them. In this context, the above questionnaire has been translated into Chinese and its proper level of validity has been reported (11). Therefore, the aim of this study was to prepare and present a valid and reliable Persian version of COMDQ so that the questionnaire can be used in Persian-speaking communities.

## Methods

The present cross-sectional study was carried out to evaluate the reliability and validity of the Persian version of COMDQ on patients referring to the Department of Oral Medicine, Babol Faculty of Dentistry in 2014‒2015 (Ethics approval code: 9237211). A total of 135 subjects were selected for the purpose of this study in two groups. Group 1 consisted of 95 patients over 18 years of age, who were literate and could easily read and write; these patients had chronic oral mucosal conditions (40 with recurrent aphthous stomatitis, 40 with oral lichen planus and 15 with pemphigus vulgaris and mucous membrane pemphigoid). These mucosal conditions were confirmed through taking a medical history, clinical examinations, hematological and histological evaluations. Group 2 consisted of 40 patients with no chronic oral mucosal conditions but with other oral mucosal conditions such as pigmented lesions, soft tissue exophytic lesions, etc. The two groups were matched in relation to age and gender.

**Preparation of the Persian version of COMDQ: **COMDQ is a tool for evaluation of the quality of life in patient with chronic oral mucosal lesions; the questionnaire is in English and consists of 26 questions in 4 domains: a) pain and functional limitations (9 questions); b) medication and treatment (7 questions); c) social and emotional status (6 questions); and d) patient support (4 questions). Based on Likert scale, there are 5 choices for each questions, from “never” (a score of zero) to “in most cases” (a score of 4); therefore, there is a score range of 0‒104 for this questionnaire, and higher scores indicate lower quality of life (4). The Persian version of COMDQ was prepared using the forward/backward method (14). First, the original version of the questionnaire was translated into Persian by two Iranian bilingual translators (with very good command of both English and Persian languages). Then, the translated Persian version was again translated into English by two English bilingual translators. Finally, these three versions, including the original COMDQ, the Persian version and the retranslated English version were evaluated by two oral medicine specialists in relation to discrepancies, no use of technical terms, accuracy and comprehensibility of the questionnaire, leading to the final approval of the Persian version of COMDQ. In the first step, the subjects included in this study received sufficient oral explanations about the study procedures and the aim of the study. Then oral consent was taken from each subject and those who were unable to understand the questionnaire were excluded from the study. For both groups of the patients, the demographic data (age and gender), educational level, the duration of disease and two questions in relation to their “opinion on their general health” and “oral health” were recorded. To register the responses to the two questions/opinions above, the three choices of “good” “moderate” and “bad” were used. Then the COMDQ was completed by the subjects. For each chronic mucosal condition, a separate scoring system was used to determine the disease severity. To confirm the severity of oral lichen planus, pemphigus and pemphigoid in their scoring systems, the factors of lesion extent (site score) and lesion activity (activity score) were verified in each site of oral cavity and finally the total site score and activity score were figured out, which resulted in the determination of the severity of the lesion (15). Visual analog scale (VAS) was used to evaluate pain severity. In the scoring system for the recurrent aphthous stomatitis, the characteristics of the lesion were ascertained during the previous three months and the factors of the number of lesions, the duration of lesions, the frequencies of the occurrence of lesions and the areas involved were used to determine disease severity. Therefore, an index referred to as total ulcer severity score (USS) was achieved from the combination of all these factors, which confirmed the severity of factors that determined the severity of the disease (16). In addition, VAS was used to evaluate pain severity. In order to determine the reliability of the questionnaire, only the subjects in group 1 were asked to return after 2 weeks to complete the questionnaire again. Data were analyzed using SPSS 17. The reliability of the questionnaire was evaluated with Cronbach’s alpha, ICC and test/retest. One-way ANOVA, t-test, *k*^2^ and Pearson’s correlation coefficient were used to evaluate the validity of the questionnaire. Statistical significance was set at p<0.05.

## Results

A total of 135 subjects were included in the present study. Table 1 presents the demographic data of the subjects. Forty patients had oral lichen planus, 40 with recurrent aphthous stomatitis and 15 had pemphigus vulgaris and mucous membrane pemphigoid; 40 patients with other oral mucosal conditions such as melanotic macula, exophytic lesions of the oral mucosa, etc. were included in the control group. In the first stage, COMDQ was completed by all the subjects in both groups. 

**Table 1:** The demographic data of the subjects

**Condition** **Variable**	**Oral lichen planus**	**Recurrent aphthous stomatitis**	**Pemphigus vulgaris**	**Other non-chronic mucosal conditions**
**Gender **				
Male Female	34 6	22 18	11 4	32 8
Age (mean ± SD)	49.28±4.24	24.98±4.306	51.07±5.59	34.25±10.21
**Educational level**				
Low education Lower secondary education High school graduate College/university	6 12 17 5	0 1 26 13	3 8 4 0	3 6 19 12

In the second stage, only 40 patients in group 1 with chronic conditions of the oral mucous membranes completed the questionnaire once again after 14 days.

**Reliability:** To evaluate the interval consistency of the questions coefficient of the questionnaire, Cronbach’s α coefficient was estimated at 0.699. Evaluation of test-retest of the question of the questionnaire showed an overall interval consistency of 0.997 for the questions, indicating a high level of consistency between the questions. Table 2 presents Cronbach’s α coefficient, intra-class correlation coefficient of the questions and the mean and standard deviations of the questions.

**Table 2:** Test-retest, reliability, internal consistency and means and standard deviations of the questions of the Persian version of COMDQ^α^

**Domains**	**Number of questions**	**Internal consistency** **(n=135)** **Cronbach’s **	**Test-retest reliability** **(n=97)** **Intraclass Correlation Coefficient**	**Mean±SD**
Pain and functional limitations	9	0.946	0.991	¶: 16.61±7.94 †: 16.7±8.15
Medications and treatment	6	0916	0.990	¶: 11.6±5.37 †: 11.6±5.48
Social and emotional status	7	0.941	0.990	¶: 14.61±6.83 †: 14.09±7.01
Patient support	4	0.697	0.995	¶: 6.98±3.44 †: 7.03±3.45
Total	26	0.969	0.997	¶: 49.81±21.15 †: 49.43±22.36

α : Chronic oral mucosal disease questionnaire

¶ : first stage

† : second stage

Evaluation of correlation between the questions of the questionnaire between the first, the second and the third sections showed a proper level of Cronbach’s α coefficient and when each question was eliminated, there was only a minor change in Cronbach’s α coefficient. However, in the 4th section of the questionnaire, on patient support, when questions 2 and 3 were eliminated, Cronbach’s α coefficient increased; however, it remained at an acceptable level, indicating there was no need for the elimination of the questions (table 3).

**Table 3:** Analysis of reliability using Cronbach’s α coefficient and Cronbach’s α coefficient after elimination of one question

**COMDQ questions**	**Corrected item; total correction**	**Cronbach’s alpha ** **when one question was eliminated**
How much do certain types of food ⁄ drink cause you discomfort (spicy food, acidic food)?	0.854	0.967
How much does your oral condition cause you to limit the types of food ⁄drinks you consume?	0.841	0.967
How much do certain food textures cause you discomfort (rough food, crusty food)?	0.812	0.968
How much does your oral condition cause you to limit the textures of the food you consume?	0.862	0.967
How much does the temperature of certain foods ⁄ drinks cause you discomfort?	0.786	0.968
How much does your oral condition cause you to limit the temperature of the foods ⁄ drinks you consume?	0.812	0968
How much does your oral condition lead to discomfort when carrying out your daily oral hygiene routine (brushing, ﬂossing, mouthwash use)?	0.856	0.967
How much does your oral condition cause you to limit your daily oral hygiene routine (brushing, ﬂossing, mouthwash use)?	0.799	0.968
How much does your oral condition lead to discomfort when wearing a denture (false teeth)?	0.00	0971
How much do you feel you need medications to help you with activities of daily life (talking, eating etc.)?	0.825	0.968
How satisﬁed are you with the medications being used to treat your oral condition?	0.810	0.968
How concerned are you about the possible side effects of the medications used to treat your oral condition?	0.689	0.969
How much does it frustrate you that there is no single standard medications to be used in your oral condition?	0.645	0.969
How much does the use of the medications limit you in your everyday life (routine ⁄ the way you apply or take your medications)?	0.708	0.968
How much does it bother you that there is no cure for your oral condition?	0.825	0.968
How much does your oral condition get you down?	0.893	0.967
How much does your oral condition cause you anxiety?	0.850	0.967
How much does your oral condition cause you stress?	0.775	0.968
How much does the unpredictability of your oral condition bother you?	0.823	0.968
How much does your oral condition cause you to worry about the future (spread of the condition, possible cancer risk)?	0.661	0.969
How much does your oral condition make you pessimistic about the future?	0.863	0.967
How much does your oral condition disrupt social activities in your life (social gatherings, eating out parties)?	0.817	0.968
How satisfactory do you consider the information available to you regarding your oral condition?	0.696	0.969
How satisﬁed are you with the level of support and understanding shown to you by family regarding this oral condition?	0.251	0.972
How satisﬁed are you with the level of support and understanding shown to you by friends ⁄ work colleagues regarding your oral condition?	0.324	0.973
How isolated do you feel as a result of this oral condition?	0.871	0.967

**Validity:** There were no relationships between the mean COMDQ scores and age (P=0.19), educational level (P=0.56) and gender (P=0.73). Patients with severe conditions, longer duration of affliction with the conditions and more severe pain had higher COMDQ scores, which were statistically significant (table 4). Subjects with greater satisfaction with their oral health status had the least COMDQ mean scores which significantly increased with a decrease in satisfaction with oral health status (table 5). In addition, there was a significant relationship between the mean COMDQ scores and the general health status of the subjects. 

However, the mean COMDQ scores were lower in patients who did not have a good opinion about their general health compared to those who had a moderate opinion about their general health status.

**Table 4:** Validity of COMDQ ^α^ in terms of variables under study

**Variables**	**Number**	**Mean ± SD**	**Pearson’s correlation**	**P-value**
**Duration of the disease**				
Aphthous Lichen planus Pemphigus vulgaris and mucous membrane pemphigoid	135	9.25±2.93 8.03±2.74 8.53±3.06	0.320 0.453 0.336	P<0.001
**Disease severity**				
Aphthous stomatitis Lichen planus Pemphigus vulgaris and mucous membrane pemphigoid	40 40 15	29.40±9.26 16.10±10.17 21.60±12.69	0.866 0.909 0.941	P<0.001
**Pain severity**				
Aphthous stomatitis Lichen planus Pemphigus vulgaris and mucous membrane pemphigoid	40 40 15	6.15±2.78 4.23±2.66 5.27±2.12	0.622 0.969 0l852	P<0.001

α : Chronic oral mucosal disease questionnaire

**Table 5:** Relationship between general and oral health states based on their own opinion and mean COMDQ ^α^ scores

**Questions**	**Number**	**Means ± SD of COMDQ scores**	**P-value**
The individual’s opinion about his/her own oral health			
Good Moderate Bad	83 21 31	30.43±5.97 57.33±11.55 74.19±6.79	P<0.001
The individual’s opinion about his/her own general health			
Good Moderate Bad	102 14 19	35.23±12.38 75.57±10.15 72.52±2.75	P<0.001

α : Chronic oral mucosal disease questionnaire

## Discussion

Chronic involvement of the oral mucosa with inflammatory and autoimmune conditions might seriously affect the social and individual quality of life of patients, including lack of self-confidence, interpersonal relationship problems, lack of expression of opinions (17‒20). COMDQ is a specific questionnaire for the evaluation of quality of life in patients with chronic oral mucosal conditions, whose validity and reliability have been confirmed (4). Differences in language, ethnicity and culture in different communities make it difficult to evaluate validity. Individuals with different cultures might not respond to the questions on COMDDQ consistently. Therefore, attention should be given to the important fact that COMDQ be adapted to different populations with different cultures, languages, ethnicities and geographic locations. The use of standard technique for translation and validity of a questionnaire cause the questionnaire to become a suitable tool in terms of psychometrics. In the present study, COMDQ was translated using the forward/backward technique (14). so the questions were adapted without any problems. 

**Evaluation of Validity:** Social and demographic variables, including age, gender and educational level, exhibited weak correlations with mean COMDQ scores. Therefore, the Persian version of this questionnaire can be comprehensible for every age group, gender and educational level, and patients can easily complete it. The mean COMDQ scores were correlated with the opinions of individuals about their oral health; hence, individuals with a better feeling about their oral health had lower scores compared to those who did not have a good feeling about their oral health. However, COMDQ scores were properly correlated with the subjects’ satisfaction with their general health status, and did not make a distinction between the subjects who had bad and moderate opinions about their general health. As a consequence, it can be concluded that this questionnaire is specific for chronic oral mucosal lesions but it cannot be considered specific in relation to the general health. Clinical parameters of disease severity and VAS were used to evaluate the validity of the questionnaire. The results showed an increase in the mean COMDQ scores in patients with chronic conditions of the oral mucosa with an increase in disease severity, the duration of the disease and pain severity. COMDQ exhibited good correlation with clinical and objective variables. Hence, it had very high validity. In a study by Ni Riordain in 2011, only VAS was used for the evaluation of the validity of the questionnaire (4). In addition, in a different study by the same authors in 2016, VAS was used again for the same purpose (12) and the results showed high validity of the questionnaire, consistent with the present study.

**Evaluation of reliability:** In the present study, COMDQ questions exhibited proper internal consistency (Cronbach’s α = 0.969). In this context, elimination of each question did not result in a significant increase in the correlation coefficient, except for questions in the patient support section; elimination of any question in this section increased the internal consistency of the questions. This finding indicated a lower internal consistency between the questions in this section and the questions in the other sections of the questionnaire. However, it was at an acceptable level and there was no need to eliminate any question. This might be attributed to cultural differences and differences in patient support in different communities. In a study by Ni Riordain, Cronbach’s α was estimated at 0.8 (4). A study in which the reliability of the Chinese version of COMDQ was evaluated Cronbach’s α was estimated at 0.894 (11), consistent with the present study. Additionally, in the two studies mentioned, similar to the present study, the patient support section exhibited lower internal consistency with the other sections, which might reflect the fact that patients with chronic conditions of oral mucosa are not sufficiently supported. In relation to the evaluation of the reliability of test/retest of COMDQ, the correlation coefficient of the questions was estimated at 0.997, which indicated a high level of compatibility between the questions. The present study was consistent with the studies by Li and Ni Riordain, who reported an ICC value of >0.8 (4, 11). Consequently, the Persian version of COMDQ is always reliable and can exhibit reproducibility at different times because it is only under this condition that similar results at two different times for one patient can indicate that the patient’s status has not changed. 

One of the limitations in this study was the small sample size, especially those patients who had pemphigus vulgaris and mucous membrane pemphigoid; further studies are suggested with larger sample size to resolve this problem. It is suggested that this questionnaire be used in longitudinal studies for the evaluation of the effect of medications on the quality of life in patients with chronic mucosal conditions. In conclusion, it can be concluded that the Persian version of COMDQ is an accurate tool with high validity and reliability and can be used in Persian-speaking communities. This tool can be utilized for the evaluation of OHQoL in cross-sectional and longitudinal studies in different age groups.
